# Tim-3 expression and its role in hepatocellular carcinoma

**DOI:** 10.1186/s13045-018-0667-4

**Published:** 2018-10-11

**Authors:** Feifei Liu, Yanning Liu, Zhi Chen

**Affiliations:** State Key Laboratory for Diagnosis and Treatment of Infectious Diseases, The First Affiliated Hospital, College of Medicine, Zhejiang University, Collaborative Innovation Center for Diagnosis and Treatment of Infectious Diseases, 79# Qingchun Road, 6A-17, Hangzhou, 310003 China

**Keywords:** Hepatocellular carcinoma, Tim-3, Immune checkpoint blockade, Immunotherapy

## Abstract

Hepatocellular carcinoma (HCC) is one of the most common tumors in the world, and its mortality is still on the rise. Limited treatments and low chemotherapy sensitivity of HCC make new therapeutic strategies urgently needed. With the rise of immune checkpoint blockade, anti-CTLA-4 antibodies and anti-PD-1 antibodies have shown therapeutic effects in various tumors. T cell immunoglobulin mucin-3 (Tim-3), a newly discovered immune checkpoint molecule, plays a major role in the development of HCC. Tim-3 can be used to evaluate the prognosis and therapeutic effects in HCC, and Tim-3 intervention has shown anti-tumor effects in preclinical experiments. This review summarizes findings regarding Tim-3 and HCC in recent years and discusses the rationale of Tim-3 as a therapeutic target for HCC.

## Background

Hepatocellular carcinoma (HCC) is the most common primary liver cancer. It is the sixth most common tumor and the third leading cause of cancer death [1]. For decades, HCC mortality in the EU, North America, and Latin America has gradually increased, and it will continue rising until 2020 according to predictions [2]. The HCC mortality in East Asia showed appreciable declines, but it maintains at a high level [2]. HCC is a typical inflammation-driven disease, nearly 90% incidences of which develop in the context of chronic liver inflammation including fibrosis and cirrhosis. Surgical resection, transplantation, radiofrequency ablation, transarterial chemoembolization (TACE), and targeted agents (Sorafenib, Regorafenib, and Lenvatinib) have been proven to prolong survival in HCC patients [1]. Nonetheless, novel therapeutic strategies are urgently needed due to the low chemotherapy sensitivity of HCC and the limited targeted drugs for HCC.

Previous studies on the tumor microenvironment (TME) have shown its crucial role in tumor progression and prognosis [3, 4]. The immune system usually has the ability to identify and eliminate tumor cells. However, in the TME, tumors develop strategies to impair functions of the immune cells by reducing antigenicity and exploiting feedback inhibition [5]. Thus, tumor immunotherapy has been developed aiming to enhance the body’s own immune system and induce anti-tumor responses. In 2013, the journal *Science* listed tumor immunotherapy including chimeric antigen receptor (CAR)-modified T cells and immune checkpoint inhibitors as the major breakthrough of the year [6]. The success of cytotoxic T lymphocyte-associated protein 4 (CTLA-4) antibodies in the treatment of early melanoma introduced immune checkpoint molecules as emerging targets for immunotherapy. To date, six immune checkpoint inhibitors have been approved for clinical use by the Food and Drug Administration (FDA). In HCC, clinical studies have focused on the programmed cell death protein 1/programmed cell death protein 1 ligand 1 (PD-1/PD-L1) pathway and CTLA-4 pathway. The PD-1 antibody Nivolumab was proved to be efficient in phase I/II clinical trials of advanced HCC patients, with high rates of response and satisfactory safety [7]. The PD-1 antibody Pembrolizumab, PD-L1 antibody Durvalumb, and CTLA-1 antibody Ipilimumab, Tremelimumab, and others have entered phase I/II clinical trials, in combination with each other or with other targeted drugs [8]. As a newly discovered immune checkpoint molecule, T cell immunoglobulin mucin-3 (Tim-3) antibodies have curative effects in laboratory-scale studies in several tumors, and some of them have entered phase I/II clinical trials (Table 1); therefore, Tim-3 has the potential to become a new target for cancer immunotherapy.

**Table 1:** Clinical trials on anti-Tim-3 agents

NCT number	Anti-Tim-3 agents	Combining agent	Mechanism of combining agents	Patients	Diseases	Phases
03489343	Sym023	–	–	48	Metastatic cancer Solid tumor Lymphoma	Phase 1
02817633	TSR-022	–	–	627	Advanced or metastatic solid tumors	Phase 1
02608268	MBG453	PDR001	Anti-PD-1	250	Advanced or metastatic solid tumors	Phase 1 and 2
03066648	MBG453	PDR001, Decitabine	Anti-PD-1, inhibit DNA methyltransferase	70	Leukemia Leukemia, myeloid Leukemia, myeloid, acute Myelodysplastic syndromes Preleukemia Bone marrow diseases Hematologic diseases	Phase 1
03099109	LY3321367	LY3300054	Anti-PD-1	172	Solid tumor	Phase 1

## Structure and functions of Tim-3

### Gene structure and protein structure of Tim-3

The Tim-3-encoding gene *HAVCR2* is located at 5q33.2 in the human genome, a region that has been linked to asthma, allergy, and autoimmunity [9]. Tim-3 is a type I cell-surface glycoprotein, including an N-terminal immunoglobulin (Ig)-like domain, a mucin domain with O-linked glycosylation and N-linked glycosylation, a single transmembrane domain, and a cytoplasmic region with a tyrosine phosphorylation motif. Tim-3 was first identified as an immunosuppressive molecule on the surface of T helper 1 (Th1) cells [10] and was then detected on cytotoxic lymphocytes (CTLs), monocytes, macrophages, natural killer cells (NKs), and dendritic cells(DCs).

### Ligands and functions of Tim-3

Tim-3 plays a key role in inhibiting both adaptive and innate immune responses. When it comes to different functions and cell types, Tim-3 binds to specific ligands. The most studied Tim-3 ligands are galectin-9 (Gal-9), phosphatidylserine (PtdSer), high-mobility group box-1 protein (HMGB1), and carcinoembryonic antigen-related cell adhesion molecule 1 (CEACAM-1) (Table 2).

**Table 2:** Ligands and functions of Tim-3 on different immune cells

Ligands	Location of Tim-3	Functions
Gal-9	CD8+ T cells	Dysfunction and apoptosis [76]
	Th1 cells	1. Th1 cell death [12] 2. Expansion of granulocytic MDSCs and MDSC-mediated immunosuppression [72]
	Treg	Cross-regulation between Th17 and Treg cells [93]
	Monocytes	Reduced phagocytic activity and HLA-DR expression [46]
	Macrophages	M2 polarization [15]
	NK	Enhanced IFN-γ production [19]
PtdSer	Macrophages	Elimination of apoptosis cells and cross-presentation [17]
	DCs	Elimination of apoptosis cells and cross-presentation [17]
HMGB1	CD8+ T cell	Prevent HMGB1-mediated T cell activation [94]
	DCs	Interfere with the recruitment of nucleic acids into DC endosomes [18]
CEACAM1	T cells	T cell exhaustion and tolerance [13, 95]

High expression of Tim-3 on effector T cells indicates T cell exhaustion, showing inhibited proliferation and TNF-α and IFN-γ secretion. The interaction of Tim-3 and its ligand, galectin-9 (Gal-9), mediates effector T cell apoptosis through calcium-calpain-caspase-1 pathway [11, 12]. While on activated T cells, CEACAM1 and Tim-3 are co-expressed and form a heterodimer to suppress T cell function and downregulate its anti-tumor immunity [13]. Tim-3+ Tregs in tumor loci exert greater suppressor function than Tim-3− Tregs. The Tim-3+ Tregs participate in shaping tumor-immune microenvironment (TIME) through supporting development of exhausted CD8+ T cells and limiting the expansion of pro-inflammatory cytokine-secreting CD4+ and CD8+ T cells [14]. Upregulation of Tim-3 on macrophages facilitates their M2 polarization and increases IL-6 secretion, further promoting tumor growth [15]. Tim-3 appears to have conflicting effects on DCs and NKs as its entire different ligands. The immunoglobulin-like region of the TIM family specifically recognizes PtdSer [16]. Interaction of PtdSer with Tim-3 on DCs mediates elimination of apoptosis cells and cross-presentation [17], while interaction of HMGB1 and Tim-3 on DCs suppresses nucleic acid-mediated innate immune responses in tumor site [18]. Tim-3 is expressed on mature NKs, and interaction of Gal-9 and Tim-3 increases IFN-γ production in NKs [19]. But the opposite result was obtained in a study in chronic hepatitis B [20]. The Tim-3/Gal-9 pathway in TIL cells is well studied in HCC.

## Tim-3 in HCC diagnosis and prognosis

### Tim-3 polymorphisms and HCC

Nearly 6000 single-nucleotide polymorphisms (SNPs) within the TIM3 gene have been identified, and several (rs246871 [21], rs10515746 [22, 23], rs10053538 [22], rs1036199 [22], rs11742259 [23], and rs35690726 [23]) may correlate to diseases. Genotype CC of rs246871 in the TIM3 gene is associated with an increased probability of HBV-associated HCC [21]. GT+TT genotypes of rs10053538 in chronic hepatitis B (CHB) patients indicate higher susceptibility to HCC, more advanced tumor grade, and shorter OS, compared with genotype GG. Zhu et al. conducted several studies to explore the associations of rs10053538 in TIM-3 with HCC. Their study in 2012 [24] found that rs10053538 GT+TT genotypes were more frequent in HCC patients of tumor grades III and IV, comparing to that in HCC patients with grades I and II, while GG genotype showed the opposite. Another study in 2013 [25] found that rs10053538 GT+TT genotypes were associated with HCC compared with cirrhosis patients without HCC. They also correlated rs10053538 with the overall survival (OS) of a prospective cohort of HBV-related HCC patients, showing that the rs10053538 GG genotype was significantly associated with longer OS, compared with GT+TT genotypes [26]. A recent study showed that rs10053538 GT+TT genotypes were associated with increased TIM-3 expression in HCC tissues [27], possibly promoting HCC progression through T cell dysfunction and tumor-associated macrophage (TAM)-induced immunosuppression, further supporting the effect of TIM3 polymorphisms on HCC traits.

### Tim-3 as a potential prognostic biomarker for HCC

Meta-analysis has demonstrated that higher expression of Tim-3 was significantly correlated with shorter OS (seven studies, HR = 1.89; 95% CI 1.38–2.57; *P* < 0.001) and more advanced tumor stage (three studies, III/IV vs. I/II, RR = 2.02; 95% CI 1.45–2.81; *P* < 0.001) in cancer patients [28]. The aberrant expression of Tim-3 has been found in tumor cells, tumor-infiltrating T cells, Tregs, and TAMs of HCC tissue. It has been verified that Tim-3 expression is correlated with HCC outcome. Hang Li et al. [29] revealed that the higher number of Tim-3+ tumor-infiltrating T cells in HCC tissues, the shorter the survival of patients. In addition, there are positive correlations of Tim-3 expression on CD14+ monocytes with tumor grades and Tim-3 expression on TAMs with poor prognosis in HCC patients [15]. Serum soluble Tim-3 (sTim-3) level is also associated with OS in HCC patients [30]. Moreover, Tim-3 expression in peripheral blood mononuclear cells (PBMCs) may be used to predict recurrence in the therapeutic liver-resected HCC patients [31]. Recurrent HCC patients present significantly higher ratio Tim-3+ CD4+ T cells and Tim-3+CD8+ T cells in PBMCs before and after liver resection than non-recurrent HCC patients.

Tim-3 can also be applied as an indicator to judge treatment efficiency. Yttrium-90-radio embolization (Y90-RE) reduces local advanced HCC and delays disease progression. By analyzing the immune status of tumor-infiltrating lymphocytes (TILs), tumor tissues, and PBMCs at various time points, Chew et al. [32] found that Tim-3+CD8+ T cells were more abundant in PBMCs of sustained responders (SRs) both before and after Y90-RE than those in non-responders. And the Tim-3+ CD8+ T cells in SRs are able to produce pro-inflammatory cytokines when stimulated in vitro. The results indicate that Tim-3+CD8+ T cells in SRs obtain potential anti-tumor effects when appropriately stimulated by tumor antigens released under radiotherapy. The high ratio of systemic Tim-3+CD8+ T cells before Y90-RE denotes patients with a sustained response after therapy as prolonging time-to-tumor progression over 6 months.

In summary, an increase in Tim-3 expression in tumor-infiltrating T cells, TAMs, and PBMCs and serum sTim-3 level in HCC indicates poor prognosis, in the form of shorter survival, more advanced tumor grades, and higher probability of recurrence. However, the high percentage of systemic Tim-3+ T cells appears to indicate optimistic outcome for HCC patients receiving Y90-RE.

## Tim-3 in TIME and HCC cells

The tumor-immune microenvironment (TIME) acts a crucial role in tumor progression. TIME contains numerous immune cells including tumor-infiltrating T cells, TAMs, regulatory cells, and resident natural killer cells. Immunosuppression is a major feature of the TIME [4]. An immunosuppressive gradient exists across the TME, the nontumor microenvironment (NTME), and peripheral blood in primary HCC [33]. Tregs, tissue-resident memory CD8+ T cells (TRMs), and TAMs are enriched in the HCC TME. The expression of T cell exhaustion markers (PD-1, Tim-3, and CTLA-4) on Tregs and TRMs from TME is higher than those in NTME or peripheral blood.

### Tim-3 and TILs

The expression of Tim-3 was first discovered on CD4+ and CD8+ T cells. The most notable role of Tim-3 is in TILs. Tim-3 was well known as a T cell exhaustion marker to suppress CTL and effector Th1 cell function. Several studies have shown that Tim-3 levels were markedly elevated in tumor-infiltrating T cells [29, 31, 34, 35].

CD8+ T cells are the most important component of TILs that exert anti-tumor functions. Large numbers of CD8+ TILs in HCC are correlated with a fair prognosis, including improved OS, longer relapse-free survival, and delayed disease progression [36]. CD8+ cytotoxic T lymphocytes (CTLs) can directly contact and lyse target cells via perforin and granzymes or induce target cell apoptosis via Fas/FasL signaling and secretion of IFN-γ and TNF. However, CD8+ TILs separated from human HCC tissues are functionally exhausted as determined by upregulated expression of PD-1, Tim-3 [29], CTLA-4, and lymphocyte activation gene 3 (LAG-3) [31] compared with those from human CHB tissues [37], tumor-free liver tissues [29, 34], and peripheral blood [34]. Functional testing further showed that these Tim3+CD8+ TILs exhibited reduced cell proliferation (Ki67) and cell activity and the production of effector cytokines (IFN-γ, IL-2, and TNF-α), indicating low anti-tumor activity.

CD4+ T cells exert contrasting roles in HCC that range from effector cell function to regulatory cell function [38]. CD4+ Th1 cells produce high levels of IFN-γ and TNF-α upon antigen stimulation and take charge of cell-mediated immunity to intracellular pathogens and tumor cells [39], while CD4+CD25+ Tregs mediate immune suppression [40]. Tim-3 mediates Th1 cell apoptosis [10], and loss of Th1 cells promotes HCC growth [41]. Several studies have shown high expression of Tim-3 along with reduced proliferation and activation potentials of CD4+ TILs in HCC tissues [29, 34, 37, 42]. The ligand Gal-9 is expressed on tumor-infiltrating APCs. DCs express a low level of Gal-9 (10%) and TAMs express the highest level of Gal-9 in HCC tissues [34]. Li et al. [29] found that anti-Tim-3 mAb could enhance Ki67 expression and IL-2 and IFN-γ production in Tim-3+CD4+ T cells cocultured with HCC-derived Gal-9+ TAMs by blocking the interaction of Gal-9 with Tim-3. On the other hand, coculture of CD14+ monocytes with TILs induced Gal-9 expression in monocytes via IFN-γ pathways [29]. These data suggest that tumor-infiltrating T cell-derived IFN-γ induces TAMs to express Gal-9, and binding of Gal-9 with Tim-3 in turn leads to T cell dysfunction.

In addition to its expression on Th1 cells, Tim-3 is also expressed on CD4+FoxP3+ Treg cells. Upregulated Tim-3 expression levels in FoxP3+ Tregs are observed in tumor sites [43]. Yan et al. [42] isolated TILs from human HCC tissues and showed that tumor-derived Tim-3+CD4+ T cells exhibited an impaired capacity to produce IFN-γ and IL-2. Phenotypic analysis revealed that the majority of these T cells expressed high levels of CD25, Foxp3, CTLA-4, and glucocorticoid-induced tumor necrosis factor receptor (GITR), which were also shared by human Tregs. They also verified a direct interaction between Tim-3+CD4+ cells and Gal-9+ cells by fluorescence in situ hybridization, indicating in vivo crosstalk between Tim-3+ CD4+ T cells and Gal-9-expressing cells. These studies suggest that TILs and Tim-3/Gal-9 signaling participate in a feedback mechanism to downregulate anti-tumor immunity; blocking Tim-3 can restore T cell function and improve anti-tumor immunity.

### Tim-3 and TAMs

The liver has the largest population of macrophages that play a central role in clearing bacteremia and recruiting immune cells. However, TAMs, especially the M2 phenotype, act as protumoral macrophages, through inhibiting effector T cell-mediated anti-tumor immunity, stimulating angiogenesis, and promoting HCC cell growth and metastasis [44, 45]. Yan et al. [15] has explained how Tim-3 affects activation and protumoral effects of TAMs in HCC. Transforming growth factor-β (TGF-β) in the HCC microenvironment enhances the transcription of Tim-3 in TAMs. Tim-3 then promotes bone marrow-derived macrophages and peripheral monocytes to differentiate into M2-like macrophages, displaying enhanced expression of M2 markers CD206 and Arg-1 and increased IL-10 production but decreased IL-12 production. Interfering Tim-3 with the anti-Tim-3 antibody, Tim-3 siRNA or Tim-3 shRNA-expressing lentivirus has the opposite outcome. M2 macrophages upregulate Tim-3 expression and increase IL-6 production through the NF-κB pathway. IL-6 consequently fosters HCC growth, migration, and invasion. Our study [46] was consistent with the former study showing that Tim-3+ monocytes conferred typical characteristics of M2 macrophages with higher CD163 and CD206 expression and IL-10 production. These studies suggest that Tim-3 on macrophages facilitate M2 polarization, promoting HCC progression by IL-6-induced tumor growth and upregulation of anti-inflammatory cytokines. We also observed reduced Tim-3 expression on monocytes was closely related to altered phagocytic activity and HLA-DR expression in monocytes [46]. The interaction between PtdSer and Tim-3 on phagocytic cells mediates uptake of apoptotic cells [16, 17]. We can reasonably assume that Tim3-expressing macrophages fail to uptake apoptotic cells, further affecting the homeostasis of the TME.

### Tim-3 and HCC cells

In addition to modulating immune cell functions, Tim-3 also regulates the function of tumor cells directly. The expression of Tim-3 on tumor cells has been detected in various cancers, including melanoma [47], non-small-cell lung cancers [48], osteosarcoma [49], malignant pleural mesothelioma [50], clear-cell renal cell carcinoma [51], cervical cancer [52], and bladder urothelial carcinoma [53]. A further mechanistic study [54] showed that tumor cell-intrinsic Tim-3 would promote HCC development by triggering auto-secretion of IL-6 and then accelerating tumor growth through the STAT3 signaling pathway. Moreover, overexpression of Tim-3 by introducing its lentiviral-expressing particles in SMMC-7721 cell line promoted cell migration and invasion by facilitating the epithelial-mesenchymal transition (EMT) [55]. These studies indicate that Tim-3 expression in HCC cells accelerates tumor growth through auto-secretion of IL-6 and enhanced metastatic ability of HCC cells by promoting EMT. On the other side, ligands of Tim-3 are spread across the TME, possibly mediating crosstalk between HCC cells and non-parenchymal cells, further affecting the aggressive phenotype of HCC cells.

## Regulation of Tim-3 expression

Given the significance of Tim-3 in HCC, it is necessary to understand its regulatory factors.

Cytokines in connection with T cell activation are involved in induction of Tim-3 expression on T cells. IL-12 and IL-27 signaling has been demonstrated to enhance transcriptional induction of *TIM3* in CD4+ and CD8+ T cells, mainly through the STAT1/T-bet and STAT3/NFIL3 pathways, respectively [56]. IL-2, IL-7, IL-15, and IL-21 were also found to induce Tim-3 expression in human T cells [57]. It was recently reported that the expression of OX40, a vital regulator of T cell activation, represented the expansion of highly suppressive Tregs in HCC. Xie et al. [58] showed that high OX40 expression was consistent with high expression of several immune-related markers, including PD-1, PD-L1, Tim-3, and LAG-3. TGF-β plays important roles in mediating T cell suppression in B cell non-Hodgkin lymphoma. TGF-β-treated effector memory T cells express high level of Tim-3 [59]. Upregulation of Tim-3 on TILs was found in cancer patients under PD-1 blockade treatments. Further studies have demonstrated that Tim-3 upregulation after PD-1 blockade depends on the PI3K/Akt pathway [60]. 14-3-3ζ protein was reported to be highly expressed in HCC and to promote the proliferation and EMT of HCC cells [61]. Recently, it was found that 14-3-3ζ can be transmitted from HCC cells to TILs by exosome-mediated delivery [62]. Overexpression of 14-3-3ζ contributes to CD8+ T cell exhaustion by upregulating PD-1 and Tim-3 expression. Long non-coding RNA (lncRNA) also regulates Tim-3 expression. Ji et al. [63] established lncRNA and mRNA expression profiles of CD3+ T cells from blood and tissues of HCC patients and healthy volunteers by using high-throughput screening. The results showed that Lnc-Tim3 was upregulated in HCC patients, and it was negatively correlated with the percentage of IFN-γ+ CD8+ T cells in tumor-infiltrating CD8+ T cells. Further experiments showed that Lnc-Tim3 bounds to the intracellular domain of Tim-3, leading to both release and nuclear localization of Bat3. Nuclear Bat3 further enhances p300/p53/p21-mediated cell cycle arrest, promoting CD8+T cell exhaustion and survival.

Tim-3 on TAMs is also regulated by cytokines in TME. Tim-3 expression was in accordance with macrophage polarization, indicating that the factors involved in macrophage polarization may affect Tim-3 expression as well on TAMs. Yan et al. [15] revealed that TGF-β in the HCC microenvironment enhanced the transcription of Tim-3 in TAMs.

There were few reports of regulation of Tim-3 in hepatoma cells. As shown above, Tim-3 promotes tumor growth and regulates EMT of HCC cells. Cytokines that affect HCC biological behaviors may participate in regulation of Tim-3 expression, including TGF-β. RepSox is a potent, selective TGFβR-1/ALK5 inhibitor [64]. A study in acute myeloid leukemia suggested that RepSox reduced Tim-3 expression by inhibiting TGF-β signaling [65]. Given the regulation of Tim-3 on TAMs and the crosstalk between HCC cells and monocytes, we can also speculate that TGF-β may be involved in the regulation of Tim-3 in HCC cells.

These results suggest that targeting the regulatory factors of Tim-3 may also be potential strategies in the Tim-3-based HCC immunotherapy.

## New approaches targeting Tim-3

### Antibodies targeting Tim-3

In recent years, antibodies targeting the PD-1/PD-L1 axis showed favorable efficacy in several cancer types. Our laboratory has confirmed the effectiveness of anti-PD-1 mAb in improving antiviral T cell responses. The CheckMate 040 trial has assessed the safety and clinical benefits of Nivolumab (an anti-PD-1 mAb) in patients with advanced HCC, showing convinced efficacy and fewer side effects. More than 30 clinical trials on PD-1/PD-L1 inhibitors and PD-1-activated cytokine-induced killer cells are in recruitment phases, combining with TACE, radiotherapy, and targeted drugs, among others. However, there is an increasing incidence of resistance to PD-1/PD-L1 blockade. Other studies [66] have found that blockade of PD-1 increased the expression of other immune checkpoint molecules on tumor-infiltrated immune cells, including Tim-3, CTLA-4, and LAG-3. Co-expression of PD-1 and Tim-3 on CD8+ T cells that accumulate in the TME has been observed in several tumor types [67, 68], and combined inhibition of both pathways has a synergistic anti-tumor effect [66, 69].

We retrieved and summarized patents on Tim-3, most of which are new designed anti-Tim-3 antibodies, bi-specific antibodies against Tim-3 and PD-1, and combinations of anti-Tim-3 antibodies and anti-PD-1 antibodies. Anti-Tim-3 antibodies have displayed anti-tumor efficacy in preclinical studies [29, 34, 42, 70] (Table 3). Anti-Tim3 monotherapy has been demonstrated to inhibit tumor progression in some tumor types. Ngiow et al. found that anti-Tim3 antibodies required the presence of CD4+ T cells and IFN-γ-expressing CD8+T cells to trigger anti-tumor immunity [71]. WT3 sarcoma in mice can be inhibited by anti-Tim3 RMT3-23 at a dose of 250 μg, injected at days 3 and 11 of WT3 inoculation. This discovery hinted at the prophylactic and therapeutic activity of anti-Tim3 RMT3-23 against sarcoma. Anti-Tim3 RMT3-23 exhibited anti-tumor efficiency in mice bearing MC38 colon adenocarcinoma, CT26 colon adenocarcinoma, and WTMCA2 fibrosarcoma as well. Dardalhon’s study showed monotherapeutic efficacy of anti-Tim-3 5D12 in EL4 lymphoma [72]. However, anti-Tim3 monotherapy encountered obstacles in some cancer types, including murine glioma [73], MCA-induced sarcoma [71], and ID8 ovarian cancer models [74]. Nevertheless, there is always a way out. Researchers found the upregulation of other immune checkpoints after blockade of Tim-3, such as PD-1 [71] and CTLA-4 [60]. Co-expression of Tim-3 and PD-1 was found to be a biomarker of ICB resistance [60]. Dual Tim-3 and PD-1 blockade [67, 69] or even combining anti-Tim-3, anti-PD-1, and anti-CTLA-4 [71] shows synergistic anti-tumor effects in several cancer types, more effective than any monotherapy. Co-blockade of TIM-3 and its ligand CEACAM1 also leads to enhanced anti-tumor immunity and improved elimination of tumors in mouse colorectal cancer models [13]. As a member of the TNFR superfamily, CD137 stimulation in CD8+ T cells promotes their proliferation, Th1-type cytokine production, and T cell survival [75]. Guo and colleagues [74] combined anti-Tim-3 RMT3-23 and CD137 activation mAb lob12.3 and proved combination of the two synergistically inhibits ID8 ovarian cancer. Anti-Tim-3 in combination with DNA methyltransferase inhibitor Decitabine has entered a phase I clinical trial. Anti-Tim-3 also increased efficacy of the chemotherapy drug cyclophosphamide in a mouse CT26 colon tumor model [76]. Tim-3 blockade combined with stereotactic radiosurgery (SRS) improved survival in glioma-burden mice more than anti-Tim-3 RMT3-23 monotherapy, and the triple therapy with anti-TIM-3, anti-PD-1, and SRS led to 100% survival [73].

**Table 3:** Preclinical studies targeting Tim-3

	Anti-Tim-3 agents	Combining agents	Mechanism of combining agents	Diseases
Anti-Tim-3 monotherapy	RMT3-23	–	–	Sarcoma, colon adenocarcinoma, fibrosarcoma [71]
	5D12	–	–	Lymphoma [72]
	Tim-3Apt	–	–	Colon carcinoma [77, 78]
Combination of anti-Tim-3 agents with other agents	RMT3–23	Anti-PD-1 antibody (clone: 29F.1A12)	Anti-PD-1	Lung cancer [66]
	RMT3–23	RMP1-14	Anti-PD-1	Melanoma, colon carcinoma [66]
	RMT3–23	UC10-4F10	Anti-CTLA-4	Melanoma [66]
	RMT3–23	RMP1-14 and UC10-4F10	Anti-PD-1 and anti-CTLA-4	Melanoma [66]
	mTim-3 hFc	Anti-PD-1 antibody (clone: MIH7)	Anti-PD-1	Acute myelogenous leukemia (AML) [67]
	Anti-TIM-3 antibody (clone: 8B.2C12)	cc1	Anti-CEACAM1	Colorectal cancer [13]
	RMT3–23	Therapeutic anti-CD137 (clone: lob12.3)	Agonistic anti-CD137 antibody	Ovarian cancer [74]
	Tim-3Apt	RMP1-14	Anti-PD-1	Colon carcinoma [77]
	Tim-3Apt	Anti-PDL1 antibody (clone: 10F.9G2)	Anti-PDL1	Colon carcinoma [78]
Combination of anti-Tim-3 agents with SRS	RMT3-23	SRS	Radiotherapy	Glioma [73]
	RMT3-23	Anti-PD-1 antibody, SRS	Anti-PD-1, radiotherapy	Glioma [73]

### Aptamers binding to Tim-3

Considering that monoclonal antibody production is troublesome and costly, developing other forms of inhibitors is an alternative strategy. Aptamers are single-stranded or peptide molecules that bind to specific target molecules. They undergo selection through a complex combinatorial process called Systematic Evolution of Ligands by Exponential Enrichment (SELEX). Gefen et al. [77] isolated a nuclease-resistant aptamer binding to Tim-3 with high affinity and specificity. The trimer-form ligand efficiently blocks the interaction of Tim-3 and Gal-9, enhancing the proliferation and anti-tumor cytokine secretion of Tim-3+T cells. The anti-tumor effects of Tim-3 aptamer were demonstrated to be superior to anti-Tim-3 monoclonal antibody both in vivo and in vitro. Tim-3 non-antigenic oligonucleotide aptamer (Tim-3Apt), identified by Hervas-Stubbs and his team [78], also displayed antagonist capacity on TIM3-expressing lymphocytes by binding to the extracellular domain of Tim-3 with high affinity and specificity. Combination of this Tim-3Apt and PDL1-blockade showed synergistic anti-tumor effects in a mouse colon carcinoma model. Selected aptamers can be chemically synthesized and exhibit great malleability, low antigenicity, and high penetration rate when compared with monoclonal antibodies, making Tim-3Apt a potential substitute for anti-Tim-3 mAb.

### Cellular therapy

Cellular therapy has always been an integral part of cancer therapy. With the maturation of gene editing technology, gene-edited autologous immune cells aid cellular therapy. Su et al. [79] generated PD-1-disrupted CTL by the CRISPR-Cas9 system and demonstrated superior cytotoxicity of these PD-1-disrupted CTLs against EBV-positive gastric cancer cells. Editing of Tim-3 or even editing of multi immune checkpoints is also feasible.

**Fig. 1:**
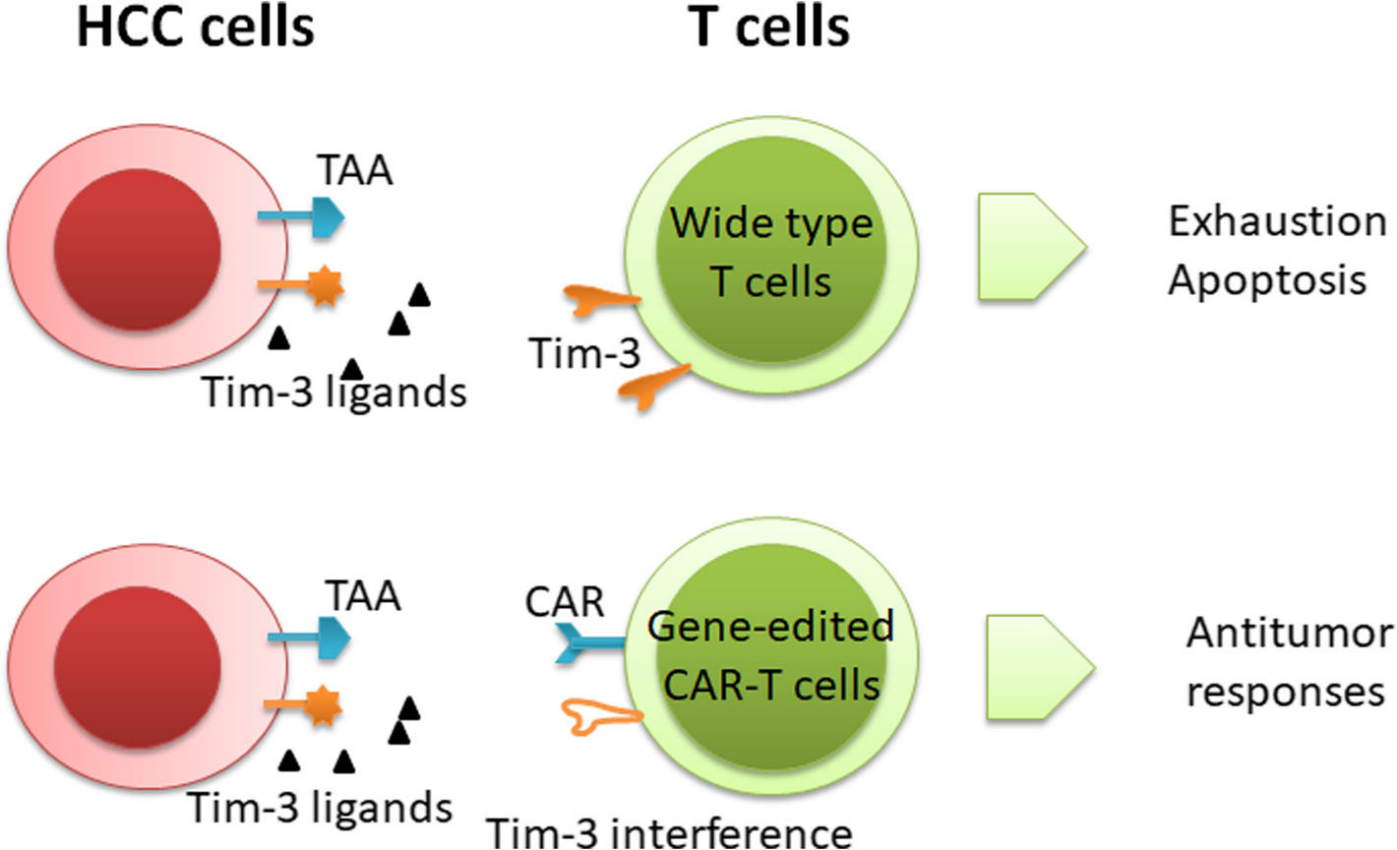
Pattern diagram of gene-edited CAR-T cells. CAR-T cells express CAR molecules against tumor-associated antigens (TAAs), which mediate specific localization and elimination of tumor cells by interacting with the TAA expressing on tumor cell surface. HCC cells are able to express ligands of immune checkpoints. The interaction of immune checkpoints and ligands leads to T cell exhaustion and apoptosis, which induces dysfunction and apoptosis of CAR-T cells as well. The immune checkpoint molecules on gene-edited CAR-T cells can be knocked out with CRISPR/cas9 system, which enables the gene-edited CAR-T cells to specifically recognize HCC cells, conduct anti-tumor responses, and avoid CAR-T cell exhaustion due to immune checkpoint pathways

The great breakthrough that chimeric antigen receptor (CAR) T cells made in blood tumors ignited researchers’ enthusiasm for cellular therapy [80]. Anti-CD19 CAR-T cells were demonstrated to be effective in the treatment of B cell leukemia and lymphoma [81]. A growing number of clinical trials of CAR-T cells are being conducted [82]. The structure of CAR molecule has been optimized to augment T cell activation and mobilize innate immune cells [83], and the targets of CAR molecules are being extensively explored. However, the TME presents barriers to the successful application of CAR-T by inhibiting T cell immunity within tumors including immune checkpoints [84]. Combining immune checkpoint inhibitors and CAR-T cells showed excellent anti-tumor immunity both in preclinical experiments [85–87] and in clinical trials [88], laying the foundation for combination of ICB and CAR-T technology. PD-1 interference by anti-PD-1 antibodies, PD-1 shRNAs, or a PD-1 dominant negative receptor breaks the suppression of CAR-T cells by tumor cells [85]. Gene-edited CAR-T cells were extensively studied. Suarez et al. [89] generated CAR-T cells targeting anti-carbonic anhydrase IX and secreting anti-PD-L1 antibodies, effectively suppressing renal cell carcinoma in a humanized mouse model. Rupp et al. [90] developed PD-1-deficient anti-CD19 CAR-T cells by combining Cas9 ribonucleoprotein (Cas9 RNP)-mediated PD-1 gene editing and CAR-expressing-lentiviral transduction, showing improved therapeutic efficacy against leukemia. CAR-T combining disruption of Tim-3 or multi immune checkpoint molecules is worth trying (Fig. 1). Anti-GPC3 CAR-T of an alternative CAR targeting molecule suppressed HCC in patient-derived xenograft models [91]. CAR-T targeting GPC3, AFP, epithelial cell adhesion molecule (EpCAM), CD133, and mucin1 has been included in clinical trials [82].

In summary, targeting Tim-3 with anti-Tim3 agents (anti-Tim-3 antibodies and Tim-3Apt) and in combination with other immune checkpoint inhibitors, CD137 agonists, and chemotherapy agents and combination with radiotherapy shows excellent anti-tumor effects in preclinical studies. These results suggest that targeting molecules involved in Tim-3 signaling with the above treatment strategies is feasible, including OX40, TGF-β, IL-2, and other cytokines [92]. Gene-edited CAR-T cells also make cellular therapy targeting Tim-3 possible.

## Challenges and prospects

ICB has achieved excellent results in preclinical and clinical treatment of tumors. However, several challenges still need to be overcome, including the drug safety, pharmaceutical technology, and the selection of treatment regimens.

Although Tim-3 is an immune checkpoint molecule, it is ubiquitously expressed in the human body, unlike the limited expression of PD-1 on exhausted T cells. Therefore, systemic application of anti-Tim-3 antibody may generate more substantial side effects. The safety and side effects still require plenty of large animal experiments and clinical trials to further examine. Meanwhile, improving the targeting of anti-Tim-3 antibody is urgently needed. The direct injection of anti-Tim-3 antibody into tumors is a method to improve targeting, requiring the combination of anti-Tim-3 antibody with transcatheter arterial embolization (TAE) or TACE. Bispecific antibodies can bind target cells and mediate antibody-dependent cell-mediated cytotoxicity (ADCC), and these two antibodies can be designated as Tim-3 antibodies and TIL-specific antibodies or tumor-associated antigen (TAA)-specific antibodies to restore immune cell function or inhibit tumor cell growth and invasion specifically.

As for the development of the Tim-3 monoclonal antibody, there may be problems of high immunogenicity, high cost, and limited plasma concentration that will significantly limit clinical application. These problems could be solved through technology upgrades or discovering highly specific and efficient Tim-3 inhibitors like Tim-3Apt. To choose appropriate immunotherapy regimens, serial measurements of multiple checkpoints will be necessary to better understand the status of the TIME. Studies have revealed that applying targeted drugs to HBV/HCV-related HCC may cause HBV/HCV activation and hepatitis, further interrupting the progress of anti-cancer treatment. Concerning this issue, the combination and timing of antiviral drugs should be taken into account when performing ICB therapy in HBV/HCV-related HCC.

## Conclusion

**Fig. 2:**
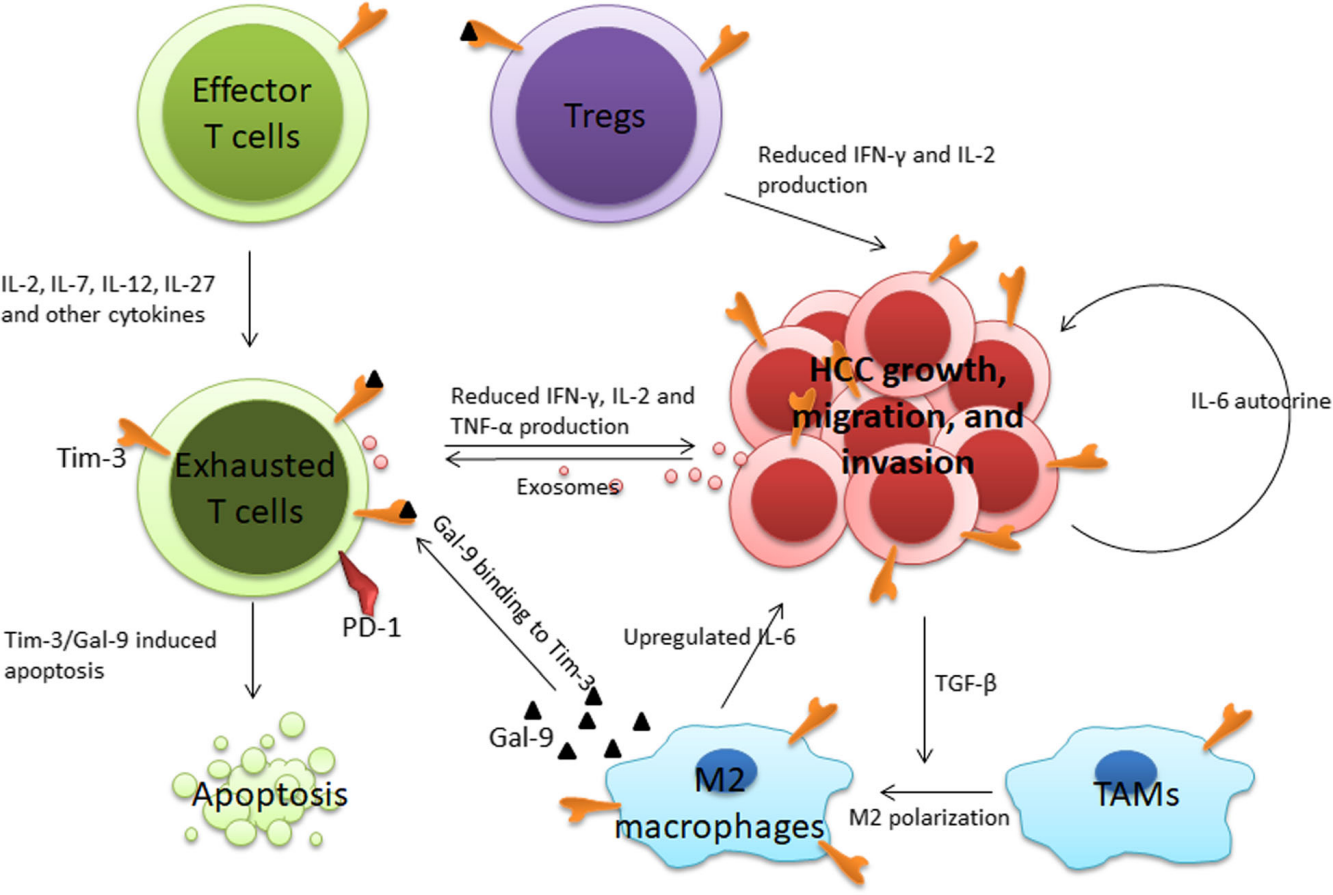
Regulation and functions of Tim-3 in HCC. Cytokines, such as IL-2, IL-7, IL-12, IL-17, TGF-β, and tumor-derived exosomes induce Tim-3 expression in T cells. Tim-3+ T cells present exhaustion phenotypes and reduced production of IFN-γ, IL-2, and TNF-α, indicating impaired anti-tumor immunity. Binding of Gal-9 to Tim-3+ effector T cells further mediates effector T cell apoptosis. Tim-3+ Tregs exert greater suppressor functions, producing reduced IFN-γ and IL-2 as well. Gal-9-expressing cells, including TAMs and DCs, are involved in the interaction of Gal-9 with Tim-3, further leading to Tim-3+ T cell exhaustion and apoptosis. HCC-derived TGF-β upregulates Tim-3 expression on TAMs and Tim-3 overexpression then facilitates M2 polarization of TAMs, further promoting HCC growth, migration, and invasion by the IL-6 pathway. Tim-3 on HCC cells promotes HCC proliferation, migration, and invasion in an IL-6 autocrine manner

Tim-3 is an immune checkpoint molecule that plays a vital role in the development of HCC. The high expression of Tim-3 in HCC tissues often indicates poor prognosis. Tim-3 inhibits anti-tumor immunity through mediating effector T cell exhaustion and apoptosis, enhancing Treg-mediated immunity suppression, and facilitating TAMs M2 polarization. Tim-3 on HCC cells also promotes HCC proliferation, migration, and invasion in an IL-6 autocrine manner (Fig. 2). Targeting Tim-3 shows anti-tumor efficiency in preclinical studies. The combination with other immune checkpoint inhibitors, CD137 agonists, and chemotherapy agents and combination with radiotherapy shows synergistic anti-tumor effects in tumor mouse models. Antibodies, aptamers, and gene-edited immune cells targeting Tim-3 and related pathways are research directions with great potential. Therefore, Tim-3 has excellent development prospects for the diagnosis and treatment of HCC.

## Abbreviations

ADCC: Antibody-dependent cell-mediated cytotoxicity
APC: Antigen-presenting cell
CAR: Chimeric antigen receptor
CAR-T: Chimeric antigen receptor-modified T cell
Cas9 RNP: Cas9 ribonucleoprotein
CEACAM-1: Carcinoembryonic antigen-related cell adhesion molecule 1
CRISPR/cas9: Clustered regularly interspaced short palindromic repeats/cas9 system
CTLA-4: Cytotoxic T lymphocyte-associated protein 4
CTLs: CD8+ cytotoxic T lymphocytes
DC: Dendritic cell
EMT: Epithelial-mesenchymal transition
EpCAM: Epithelial cell adhesion molecule
Gal-9: Galectin-9
GITR: Glucocorticoid-induced tumor necrosis factor receptor
HCC: Hepatocellular carcinoma
HMGB1: High-mobility group box-1 protein
ICB: Immune checkpoint blockade
IFN-γ: Interferon-γ
LAG-3: Lymphocyte activation gene 3
lncRNA: Long non-coding RNA
NK: Natural killer cell
NTME: Nontumor microenvironment
OS: Overall survival
PBMC: Peripheral blood mononuclear cell
PD-1: Programmed cell death protein 1
PD-L1: Programmed cell death protein 1 ligand 1
PtdSer: Phosphatidylserine
SELEX: Systematic Evolution of Ligands by Exponential Enrichment
shRNA: Short hairpin RNA
siRNA: Small interfering RNA
SNPs: Single-nucleotide polymorphisms
SR: Sustained responder
SRS: Stereotactic radiosurgery
sTim-3: Soluble Tim-3
TAA: Tumor-associated antigen
TACE: Transcatheter arterial chemoembolization
TAE: Transcatheter arterial embolization
TAM: Tumor-associated macrophage
TGF-β: Transforming growth factor-β
Th1: Helper T cell 1
Th17: Helper T cell 17
TILs: Tumor-infiltrating lymphocytes
Tim-3: T cell immunoglobulin mucin-3
Tim-3Apt: Tim-3 non-antigenic oligonucleotide aptamer
TIME: Tumor-immune microenvironment
TME: Tumor microenvironment
TNF: Tumor necrosis factor
TNFR: Tumor necrosis factor receptor
Treg: Regulatory T cell
TRMs: Tissue-resident memory CD8+ T cells
Y90-RE: Yttrium-90-radioembolization
